# Pyrolysis Kinetic Properties of Thermal Insulation Waste Extruded Polystyrene by Multiple Thermal Analysis Methods

**DOI:** 10.3390/ma13245595

**Published:** 2020-12-08

**Authors:** Ang Li, Wenlong Zhang, Juan Zhang, Yanming Ding, Ru Zhou

**Affiliations:** 1 College of Power Engineering, Naval University of Engineering, 717 Jiefang Ave, Qiaokou District, Wuhan 430032, China; sirius027lai@cug.edu.cn; 2 Faculty of Engineering, China University of Geosciences, 388 Lumo Rd, Hongshan District, Wuhan 430074, China; zhangjuan@cug.edu.cn (J.Z.); dingym@cug.edu.cn (Y.D.); 3 Jiangsu Key Laboratory of Urban and Industrial Safety, College of Safety Science and Engineering, Nanjing Tech University, 30 Puzhu Rd, Pukou District, Nanjing 211816, China

**Keywords:** extruded polystyrene, pyrolysis, kinetic model, thermal degradation, reaction mechanism

## Abstract

Extruded polystyrene (XPS) is a thermal insulation material extensively applied in building systems. It has attracted much attention because of outstanding thermal insulation performance, obvious flammability shortcoming and potential energy utilization. To establish the reaction mechanism of XPS’s pyrolysis, thermogravimetric experiments were performed at different heating rates in nitrogen, and multiple methods were employed to analyze the major kinetics of pyrolysis. More accurate kinetic parameters of XPS were estimated by four common model-free methods. Then, three model-fitting methods (including the Coats-Redfern, the iterative procedure and masterplots method) were used to establish the kinetic model. Since the kinetic models established by the above three model-fitting methods were not completely consistent based on different approximations, considering the effect of different approximates on the model, the reaction mechanism was further established by comparing the conversion rate based on the model-fitting methods corresponding to the possible reaction mechanisms. Finally, the accuracy of the above model-fitting methods and Particle Swarm Optimization (PSO) algorithm were compared. Results showed that the reaction function *g*(*α*) = (1 − *α*)^−1^ − 1 might be the most suitable to characterize the pyrolysis of XPS. The conversion rate calculated by masterplots and PSO methods could provide the best agreement with the experimental data.

## 1. Introduction

Energy has played a significant role in promoting economic growth. However, the current global energy problem is already one of the main problems restricting sustainable development [1]. Besides, the issue of energy consumption in buildings is increasingly prominent, and buildings account for more than 30% of global energy consumption [2]. Therefore, many countries are committed to improving energy efficiency, especially in buildings [3]. Therein, the thermal insulation material is one of the most effective approaches to economize energy [4]. Currently, organic polymer foam insulation board [5], such as extruded polystyrene (XPS), is extensively applied as insulation material in building insulation systems due to its outstanding performance, such as low thermal conductivity, lightweight and so on [6].

However, due to the low thermal stability, XPS is easily affected by high temperature and intense solar radiation [7], which damages the characteristics of XPS. In addition, XPS is flammable, more and more building fires can be attributed to XPS, such as Grenfell Tower [8]. The severity of the fire is related to the spread speed, and one of the main reasons for the rapid spread is that the insulation material is easy to ignite, and the degradation products containing gaseous fuel contribute to combustion [9], such as styrene monomer and oligomers [10]. Since pyrolysis is a key component in the combustion process, so it is imperative to study the thermal decomposition characteristic of XPS for predicting the growth of fire [11]. On the other hand, because of the large amount of XPS waste, the issue of disposal of the waste is becoming increasingly urgent [12]. However, improper processing of waste could lead to a series of problems, such as waste of resources [13], environmental contamination [14], fire hazard [15] and so on. Among the commonly-used methods of solid waste treatment, pyrolysis is expected to be a meaningful energy conversion method that can convert solid waste into fuel [16] and recover useful chemicals [17]. What is more, pyrolysis plays an important role in waste plastics for energy recovery [18,19]. Especially, valuable feedstock and fuel are obtained from the pyrolytic process of waste plastics [20,21]. In recent years, the thermal degradation of solid waste has attracted increasing attention owing to the potential to substitute traditional fossil fuels [22].

As discussed above, the knowledge of pyrolysis characteristics of XPS not only is closely related to the fire risk but also facilitates the recycling of XPS waste. There have been many studies on the pyrolysis characteristics of XPS. Jiao et al. [23,24] investigated the pyrolysis of XPS with expanded polystyrene and polyurethane foam and further studied its pyrolysis characteristics in different environments. Jiang et al. [25] studied the pyrolysis behavior of XPS waste to obtain the kinetic model and reconstruct the function of the model. As a result of these studies, the pyrolysis characteristics of XPS can be further understood.

In addition, many researchers pointed out that different approximations used in the calculation of kinetic parameters would affect their accuracy, which brought errors in the reaction mechanism. For example, Farjas et al. [26] noted that the accuracy of the integral isoconversional method was linked to approximations. Vyazovkin et al. [27] also indicated that an error occurred in the calculation of the activation energy because of approximations. However, the reaction mechanism of XPS’s pyrolysis is commonly established by coupling the model-free and model-fitting methods [25], which rarely considers the influence of the approximations. Therefore, the purpose of this study is to establish the reaction mechanism of XPS’s pyrolysis by multiple methods while considering approximations and find which method can reflect the reaction process with the highest accuracy. Besides, the accurate pyrolysis kinetics of XPS can be used for large-scale fire simulations, such as the Fire Propagation Apparatus [28] and Cone Calorimetry [29]. Furthermore, they contribute to guiding the reactor design [30].

In the current study, thermogravimetric experiments were performed to obtain the pyrolysis characteristics of XPS in nitrogen. More accurate kinetic parameters were estimated by multiple typical model-free methods (such as Flynn–Wall–Ozawa, Starink, Distributed Activation Energy Model and Tang method). Then, model-fitting methods (including Coats–Redfern, the iterative procedure, and masterplots method) were used to establish the kinetic model of XPS. Considering the effect of the approximations on the model, the pyrolysis reaction mechanism was further established by comparing the conversion rate based on the model-fitting methods corresponding to the possible reaction mechanisms. Finally, the accuracy of the above model-fitting methods and Particle Swarm Optimization (PSO) algorithm were compared.

## 2. Materials and Methods

### 2.1. Materials

The XPS employed in this study was milled to powder and then put into an oven to lower the water content before testing. The element analysis was performed by Vario EL cube. The results showed that elements C, H, N and S on a dry basis were 71.60%, 6.43%, 1.24% and 0.918%, respectively.

### 2.2. Thermogravimetric Measurements

Thermogravimetry experiments were conducted by TA Instruments on SDT Q600 (New Castle, DE, USA). The 6 mg sample was evenly placed in an aluminum oxide crucible during the experimental temperature 300–1000 K. Nitrogen was a purge gas, and its flow rate was 100 mL/min. In order to be close to the heating rates of real fires, the heating rates of 5 K/min, 20 K/min, 40 K/min, 60 K/min and 80 K/min were selected.

### 2.3. Pyrolysis Kinetics

Thermogravimetry provides an ideal environment for the degradation of the small solid sample in which the atmosphere and heating rates can be well controlled [31]. The solid reaction rate during the decomposition can be written as

(1) $$\frac{d\alpha }{dt}=k\left(T\right)f\left(\alpha \right)$$

where *f*(*α*) denotes the differential function. *t* is time, *α* represents conversion rate, and *k*(*T*) denotes a constant with temperature *T*. *α* and *k*(*T*) can be defined as follows:

(2) $$\alpha =\frac{m_{0}-m_{t}}{m_{0}-m_{\infty }}$$

(3) $$k\left(T\right)=A\exp\left(\frac{-E_{a}}{RT}\right)$$

Three types of *m* (*m*_0_, *m_t_* and *m*_∞_) stand for initial, transient and final mass, respectively. *E_a_* represents activation energy, *A* refers to the pre-exponential factor, and *R* means the universal gas constant.

Considering the linear relationship between temperature and the heating rate (*β*), *β* = *dT*/*dt*, the reaction rate can be substituted as

(4) $$\frac{d\alpha }{dT}=\frac{A}{\beta }f\left(\alpha \right)\exp\left(\frac{-E_{a}}{RT}\right)$$

Then the integral function *g*(*α*) is expressed as

(5) $$g\left(\alpha \right)=\int_{0}^{\alpha }\frac{d\alpha }{f\left(\alpha \right)}=\frac{A}{\beta }\int_{T_{0}}^{T}\exp^{-(\frac{E_{a}}{RT})}dT\approx \frac{AE_{a}}{\beta R}P\left(x\right)$$

where *x* = *E_a_*/*RT*. *P*(*x*) indicates the temperature integral. There are many approximations of *P*(*x*) introduced in the literature [26], and they can be represented as

(6) P(x)≈exp(−1.0518x−5.330)

(7) $$P\left(x\right)≅\frac{\exp(-1.0008x-0.312)}{x^{1.92}}$$

(8) −ln(P(x))≈0.377739+1.894661lnx+1.00145x

(9) $$P\left(x\right)\approx \frac{\exp\left(-x\right)}{x^{2}}\left(\frac{x^{5}+40x^{4}+552x^{3}+3168x^{2}+7092x+4320}{x^{6}+42x^{5}+630x^{4}+4200x^{3}+12,600x^{2}+15,120x+5040}\right)$$

(10) $$P\left(x\right)=\frac{\exp\left(-x\right)}{x^{2}}\times \left(1+\frac{2!}{-x}\right)$$

### 2.4. Methods

Model-free and model-fitting methods are common methods for analyzing kinetics. For the model-free methods, the advantage is that the activation energy can still be calculated when the reaction mechanism is not known [32], while the model-fitting methods can determine the reaction mechanism and obtain a set of corresponding kinetic parameters based on the reaction mechanism [25]. Common solid reaction mechanisms are listed in Table 1.

#### 2.4.1. Model-Free Methods

Two forms of model-free methods, namely differential and integral conversion methods, are widely employed [31]. However, Vyazovkin et al. [31] noted that the differential methods were not more accurate than the integral methods. Therefore, in the current study, the integral isoconversional methods are applied. These integral isoconversional methods are different depending on the approximations.

##### Flynn–Wall–Ozawa Method (FWO)

The FWO method [35,36] estimate *E_a_* by the slope of the linear plot of ln*β* versus 1/*T*. The equation can be written as Equation (11) based on the approximation of Equation (6).

(11) $$\ln\beta =\ln\left(\frac{AE_{a}}{Rg\left(\alpha \right)}\right)-5.331-1.052\left(\frac{E_{a}}{RT}\right)$$

##### Starink Method

Similar to the FWO method, the Starink [37] method is also employed to calculate the *E_a_* by the slope (ln*β*/*T^1.92^* versus 1/*T*). The equation based on Equation (7) can be expressed as

(12) $$\ln\frac{\beta }{T^{1.92}}=\ln\left(\frac{AE_{a}}{Rg\left(\alpha \right)}\right)-0.312-1.0008\left(\frac{E_{a}}{RT}\right)$$

##### Tang Method

Besides, the Tang [38] method adopts Equation (8), which can be expressed as Equation (13) to estimate the *E_a_* by the slope of ln*β*/*T*^1.894661^ versus 1/*T*.

(13) $$\ln\frac{\beta }{T^{1.894661}}=\ln\left(\frac{AE_{a}}{Rg\left(\alpha \right)}\right)+3.635041-1.89466\ln E_{a}-1.00145\left(\frac{E_{a}}{RT}\right)$$

##### Distributed Activation Energy Model Method (DAEM)

The DAEM method is an extensively accepted method to calculate the pyrolysis kinetics of complex materials [39]. Its simplified function is presented in Equation (14) based on Equation (9) [40].

(14) $$\ln\frac{\beta }{T^{2}}=\ln\left(\frac{AR}{E_{a}}\right)+0.6075-\frac{E_{a}}{RT}$$

As shown in Equation (14), both *E_a_* and ln*A* can be obtained from the slope and intercept by plotting ln*β*/*T*^2^ versus 1/*T*.

#### 2.4.2. Model-Fitting Methods

The model-fitting methods match the theoretical kinetic models according to the thermogravimetric experimental data, and the corresponding model is determined as the kinetic model of solid when the theoretical value of the kinetic parameters is best fitted with the experimental value [41]. The common model-fitting methods contain the Coats–Redfern method (CR), the iterative procedure and masterplots method and so on. Especially, Ding et al. obtained the woody biomass pyrolysis kinetic model through the optimization algorithms, such as Shuffled Complex Evolution (SCE) [42], PSO [43] and Genetic Algorithm method [44]. Therefore, from the perspective of establishing the kinetic model, optimization algorithms can also be considered as a model-fitting method [43].

##### Coats–Redfern Method

The CR method [45] is one of the most commonly-used model-fitting methods, and the equation can be expressed as Equation (15) using the approximation of Equation (10).

(15) $$\ln\frac{g\left(\alpha \right)}{T^{2}}=\ln\left(\frac{AR}{\beta E_{a}}\right)-\frac{E_{a}}{RT}$$

Kinetic parameters (*E_a_* and ln*A*) corresponding to each reaction function *g*(*α*) are obtained by the plot of ln(*g*(*α*)/*T^2^*) versus 1/*T*.

##### The Iterative Procedure

In addition, the iterative procedure [46] is also applied to determine the solid kinetic model. The expression of the iterative procedure method, namely *g*(*α*) function is written as

(16) $$\ln\left(g\left(\alpha \right)\right)=\left(\ln\left(\frac{AE_{a}}{R}\right)+\ln\left(P\left(x\right)\right)\right)-\ln\beta$$

If the kinetic model can reflect the solid pyrolysis process appropriately, there is a linear relationship between ln(*g*(*α*)) versus ln*β*, and the slope should be close to −1, and the linear correlation coefficient *R*^2^ is higher [47]. The *P*(*x*) applies to the approximation of Equation (9).

##### Masterplots Method

Masterplots method [48] is obtained by taking *α* = 0.5 into Equation (5), and it is expressed by the following equation:

(17) $$\frac{g\left(\alpha \right)}{g\left(0.5\right)}=\frac{P\left(x\right)}{P\left(x_{0.5}\right)}$$

where *x*_0.5_ = *E_a_*/*RT*_0.5_. To quantify the application of Equation (17), statistics number *F* for estimating the fitness of each model is applied, as shown in Equations (18) and (19) [25].

(18) $$S_{j}^{2}=\frac{1}{n-1}{\sum_{i=1}^{n}\left(\frac{P_{i}}{P_{0.5}}-\frac{g_{j}\left(\alpha_{i}\right)}{g_{j0.5}}\right)}^{2}$$

(19) $$F_{j}=\frac{S_{j}^{2}}{S_{\min}^{2}}$$

where *i* and *j* are conversion rate and heating rate, respectively. If *F* = 1 for each heating rate of the model, the model is regarded as a kinetic model of solid pyrolysis.

#### 2.4.3. Particle Swarm Optimization Method

The optimization algorithms have been employed to optimize kinetic parameters due to high efficiency and good accuracy, especially the reaction mechanism established reflects the process of solid pyrolysis when the kinetic parameters are globally optimal [29,49]. The fitness value of PSO can be obtained from the following formulas:

(20) $$\phi =\phi_{\alpha }+\phi_{d\alpha /dt}$$

(21) $$\phi_{\alpha }=\sum_{j=1}^{N}\left[w_{\alpha ,j}\frac{\sum_{k=1}^{n}{\left(\alpha_{\mathrm{mod},k}-\alpha_{\exp,k}\right)}^{2}}{\sum_{k=1}^{n}{\left(\alpha_{\exp,k}-\frac{1}{n}\sum_{p=1}^{n}\alpha_{\exp,p}\right)}^{2}}\right]$$

(22) $$\phi_{d\alpha /dt}=\sum_{j=1}^{N}\left[w_{d\alpha /dt,j}\frac{\sum_{k=1}^{n}{\left({d\alpha /dt}_{\mathrm{mod},k}-{d\alpha /dt}_{\exp,k}\right)}^{2}}{\sum_{k=1}^{n}{\left({d\alpha /dt}_{\exp,k}-\frac{1}{n}\sum_{p=1}^{n}{d\alpha /dt}_{\exp,p}\right)}^{2}}\right]$$

where *Φ* refers to the objective value. *α* and *dα*/*dt* denote the cumulative values of conversion rate and reaction rate, respectively. *N* and *n* indicate the number of experiments and experimental data points, respectively. *w* presents the weighted value. Subscript mod and exp are calculated values from simulations and experiments.

Suppose to search in the *D*-dimensional space of *n* particles, the position and velocity vectors of the *i*th particle are expressed as *x_i_* = (*x_i_*_1_, *x_i_*_2_, …, *x_iN_*) and *v_i_* = (*v_i_*_1_, *v_i_*_2_, …, *v_iN_*), respectively. The particle update can be obtained by the following equations:

(23) $$v_{id}^{k+1}=wv_{id}^{k}+c_{1}r_{1}\left(p_{id}-x_{id}^{k}\right)+c_{2}r_{2}\left(p_{gd}-x_{id}^{k}\right)$$

(24) $$x_{id}^{k+1}=x_{id}^{k}+v_{id}^{k+1}$$

where *i* and *k* are the number of particle and iteration, respectively. *d* indicates the search direction. *p_id_* and *p_gd_* are the optimal personal position and the global position, respectively. *c*_1_ and *c*_2_ are constants of positive acceleration that represent the individual and global properties of the swarm. *r*_1_ and *r*_2_ are random numbers from 0 to 1.

## 3. Results and Discussion

### 3.1. Thermogravimetric Analysis

**Figure 1:**
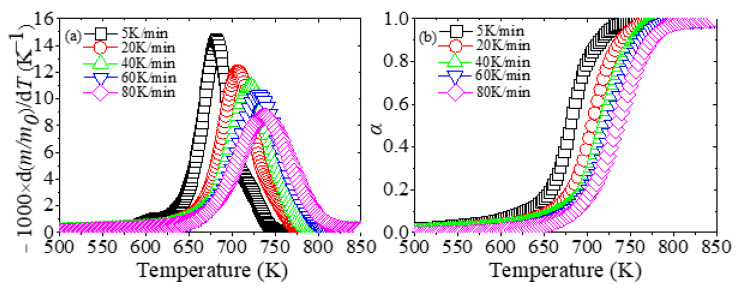
The (**a**) derivative mass loss (DTG) and (**b**) conversion rate profiles of degradation processes of extruded polystyrene (XPS) at different heating rates.

Figure 1 illustrates the derivative mass loss (DTG) and conversion rate profiles of degradation processes of XPS at different heating rates.

As shown in Figure 1, the movement of the DTG and conversion rate curves is related to the heating rates. As the heating rate increases, the reaction range is gradually delayed to a higher temperature to complete the reaction. For example, the peak temperatures *T_P_* of the DTG curves at five heating rates are 681 K, 707 K, 721 K, 731 K and 737 K. In addition, the initial decomposition temperature of XPS is 575–625 K, and the final temperature is 750–825 K, and the reaction temperature range is about 175 K. Moreover, Jun et al. [50] introduced a classic method called Coats–Redfern to calculate the kinetic parameters of expandable polystyrene and suggested that if there was just one peak in the DTG curve, it indicated that one kind of reaction occurred. Since each DTG curve has only one peak, the pyrolysis of XPS in nitrogen is a one-step reaction.

### 3.2. Kinetic Analysis by the Model-Free Methods

The activation energy *E_a_* calculated based on the FWO, DAEM, Starink and Tang methods is shown in Table 2. It is noted that the *E_a_* of Jiang et al. [12] and Jiao et al. [24] is obtained by the Kissinger–Akahira–Sunose (KAS) method. There are some reasons why the KAS method is not chosen to calculate the *E_a_* in this study, but the *E_a_* obtained by KAS in References [12,24] is compared. The *E_a_* can be obtained by the slope of the linear relationship between the heating rate *β* and temperature *T*. For the KAS method, *E_a_* is obtained through the slop of ln(*β*/*T*^2^) and 1/*T*. However, it is the same as the value of the DAEM method. Since the DAEM method has some advantages [38], the DAEM rather than KAS is used to estimate the *E_a_* in the current study. For Reference [12], FWO and KAS methods were applied to estimate the kinetics of XPS. However, the FWO method is slightly inaccurate compared with other model-free methods [31]. Furthermore, the *E_a_* calculated from the FWO method is larger than that calculated by KAS and Starink [13]. In this study, the *E_a_* is also a little larger than that of DAEM, Starink and Tang methods. Besides, the *E_a_* was only calculated by the KAS method in Reference [24]. Therefore, the calculated results obtained by the KAS method in the literature are compared.

Table 2 shows that by comparing References [12,24], there will be a difference in *E_a_*. There are many factors that affect the calculated values of kinetics, such as raw material source, heating rates, temperature, gas flow and so on [51,52]. Jiang et al. [12] selected four heating rates (5 °C/min, 10 °C/min, 15 °C/min and 20 °C/min) and conducted the thermogravimetric analysis with a gas flow of 20 mL/min in nitrogen. Furthermore, the sample weighed about 6 mg, and it was cut to powder and heated up to 800 °C. In the test of Jiao et al. [24], 4 mg particulate sample was heated to 700 °C with four heating rates (5 K/min, 10 K/min, 20 K/min and 30 K/min), and the flow rate of nitrogen was 75 mL/min. In this study, the 6 mg powdered sample was tested at heating rates (5 K/min, 20 K/min, 40 K/min, 60 K/min and 80 K/min), and the temperature was 300–1000 K, and the flow rate of nitrogen was 100 mL/min. It is noted that the calculated values of the literature are only used to compare with calculated results of this study, and they are not the boundaries of the range.

It can also be seen from Table 2 that the *E_a_* maintains constant, and the average *E_a_* is 200.4 kJ/mol (average of four methods). Furthermore, researchers [53,54] noted that if the deviation between the maximum and minimum *E_a_* was less than 20–30% of the average *E_a_*, then the *E_a_* was independent of *α*. Table 2 shows that the calculated values by four methods are less than 20% of the average *E_a_*, so the pyrolysis of XPS is a one-step reaction in nitrogen, which is also proved by Figure 1.

### 3.3. Establishment of Reaction Mechanisms

In this study, the calculated *E_a_* of the CR method at different heating rates is illustrated in Table 3. Then, it compares with that previously obtained *E_a_* using the four model-free methods. The pyrolysis reaction mechanism of XPS should be established when the average *E_a_* of the kinetic model based on the CR method is the closest to that of model-free methods [32].

Table 3 shows that the values of *E_a_* of four reaction models are closest to 200.4 kJ/mol, and their models are No. 4 (189.3 kJ/mol), No. 17 (205.2 kJ/mol), No. 18 (210.0 kJ/mol) and No. 19 (186.0 kJ/mol), respectively. Jiang et al. [25] established a pyrolysis reaction function *g*(*α*) = −ln(1 − *α*) of XPS in nitrogen. However, the *E_a_* of this reaction function is 143.0 kJ/mol by the CR method in this study, and the difference is large, which indicates reaction function *g*(*α*) = −ln(1 − *α*) is not applicable to the current study.

Since the *E_a_* is estimated through the model-free methods and the CR method in different approximations [55], and the difference corresponding to these models is very small (11.1 kJ/mol, 4.8 kJ/mol, 9.6 kJ/mol and 14.4 kJ/mol), so it cannot 100 percent determine the kinetic model of XPS in nitrogen. To improve accuracy, masterplots and the iterative procedure methods are also applied to determine possible kinetic models. The calculation results of the two methods are listed in Table 4. As presented in Table 4, the model of No. 18 is the best by masterplots method. However, the model of No. 4 is the best by the iterative procedure method.

To establish a suitable reaction mechanism, “kinetic compensation effects (KCE)” is generally accepted [56]. If the model is proper, good linear relation occurs between *E_a_* and ln *A*, as expressed in Equation (25).

(25) $$\ln A=a+bE_{a}$$

**Figure 2:**
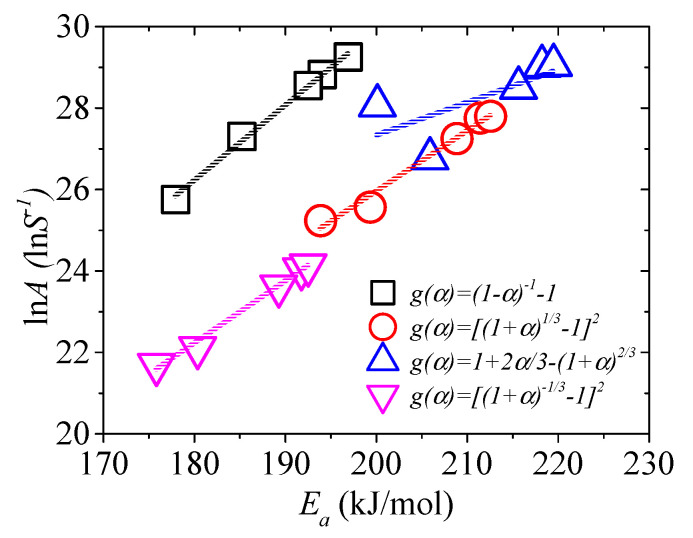
The plot of ln*A* versus *E_a_*.

The KCE of the four models is shown in Figure 2. It shows that the linear relationship for *g*(*α*) = 1 + 2*α*/3 − (1 + *α*)^2/3^ is not suitable. It also shows that other models are suitable, and the linear relationships are expressed as ln*A* = 0.184*E_a_* − 6.94 (No. 4 model, *R*^2^ = 0.995), ln*A* = 0.148*E_a_* − 3.69 (No. 17 model, *R*^2^ = 0.971), ln*A* = 0.153*E_a_* − 5.24 (No. 19 model, *R*^2^ = 0.989). Jiang et al. [25] noted that the kinetic model corresponding to the highest *R*^2^ did not mean that it was the real reaction model. Therefore, the three models mentioned above are most possibly the pyrolysis kinetic models of EPS in nitrogen.

**Figure 3:**
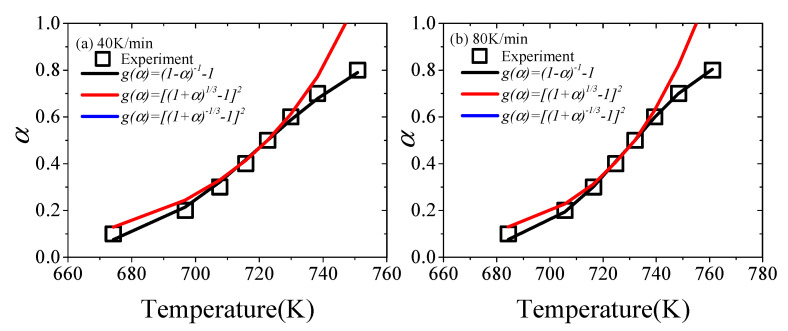
The experimental and theoretical *α* in the cases of (**a**) 40 K/min and (**b**) 80 K/min.

If the reaction mechanism of XPS’s pyrolysis is selected correctly, the reaction parameters can be in good agreement with experimental data throughout the pyrolysis process [57]. Thus, the conversion rate α of the theoretical value is fitted to the experimental data. The theoretical α of three reaction models can be estimated by model-fitting methods. The comparison of experimental and theoretical *α* at 40 and 80 K/min is shown in Figure 3.

Figure 3 shows that among the three possible reaction mechanisms, the *α* corresponding to the No. 4 model obtained by the masterplots method has a good consistency with the experimental value throughout the experiment. The *α* of the No. 17 model partially fits the experimental value in the masterplots method, but the deviation is larger. As for the No. 19 model, the deviation is the largest, and the value of *α* is negative, so it is not shown in Figure 3. Therefore, the reaction function of XPS in nitrogen is *g*(*α*) = (1 − *α*)^−1^ − 1.

It is noted that the XPS products are various, and the properties may be different. Therefore, the reaction mechanism determined as the most suitable may not completely use on the results from research shown in the literature [12,24]. For example, although this paper and Jiang et al. [25] have both studied the pyrolysis characteristics of XPS, the reaction function of Jiang et al. [25] was not suitable for this study.

### 3.4. Comparison of Multiple Kinetic Methods

**Figure 4:**
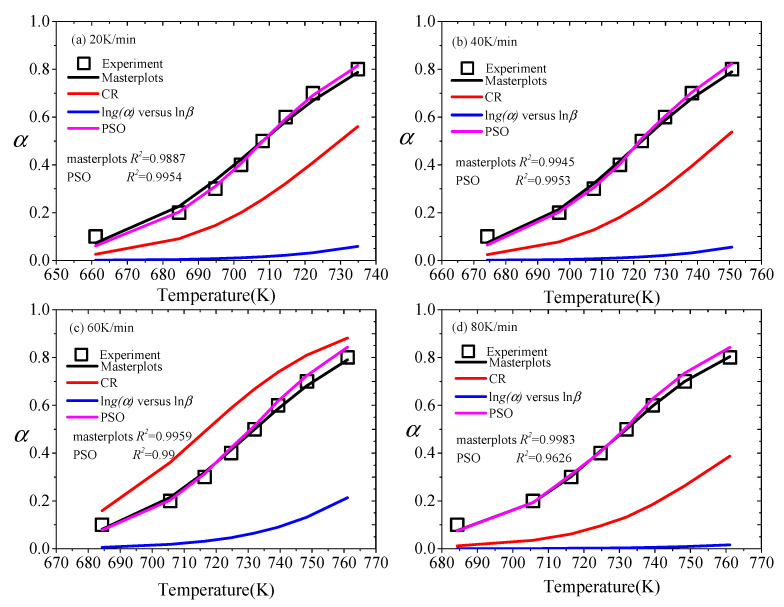
The *α* of CR, the iterative procedure, masterplots and PSO methods are compared with experimental data, (**a**) 20 K/min, (**b**) 40 K/min, (**c**) 60 K/min, (**d**) 80 K/min.

There are many methods to obtain the kinetic model of solid state. To obtain a more accurate analysis, it is necessary to compare multiple kinetic methods. As shown in Figure 4, the *α* of CR, the iterative procedure, masterplots and PSO methods is compared with the experimental value in the cases of 20, 40, 60 and 80 K/min.

Vyazovkin et al. [31] noted that the kinetic model mainly consisted of three forms by reaction profiles (*α* vs. *T*), including sigmoidal form, decelerating form and accelerating form. As presented in Figure 4, the reaction temperature range corresponding to the heating rate is different, but the trend of change is consistent. The *α* of the masterplots and PSO methods is basically consistent with the experimental value in the process of pyrolysis. The model is a decelerating model [58]. Besides, through the comparison between the masterplots method and the PSO method, it is found that their agreement with the experimental value varies slightly with the heating rates. For 20 and 40 K/min, the *α* calculated by the PSO method matches the experimental value better than that calculated by the masterplots method. However, for 60 and 80 K/min, the accuracy of the calculated value of the masterplots method is better than the PSO method. Although the CR method and the iterative procedure method are widely applied, the real pyrolysis process of XPS is not shown by them. The trend of the calculation results of the CR method is mostly accelerating, but Figure 4c is decelerating. Besides, the iterative procedure method has a larger deviation.

## 4. Conclusions

To study whether approximations affect the accuracy when establishing the reaction mechanism of XPS’s pyrolysis, which method can reflect the reaction process and have the highest accuracy among the multiple methods, the kinetic model of XPS pyrolysis was investigated from 5 K/min to 80 K/min in this study. Four model-free methods (such as FWO, DAEM, Starink and Tang method) were employed to calculate the more accurate kinetic parameters, and four kinetic methods (including CR, the iterative procedure, masterplots and PSO) were applied to estimate the conversion rate with the comparison of experimental data. The results showed that four reaction mechanisms were close if only the activation energy between model-free methods and the CR method is compared. What is more, the reaction mechanisms of XPS’s pyrolysis established via multiple kinetic methods were different. Therein, the reaction function *g*(*α*) = (1 − *α*)^−1^ − 1 might be the most suitable to characterize the pyrolysis of XPS in nitrogen. Furthermore, masterplots and PSO methods were more accurate than the CR and the iterative procedure methods. The pyrolysis kinetics of XPS can be used for large-scale fire simulations, such as the Fire Propagation Apparatus and Cone Calorimetry. Furthermore, they are important guidance for reactor design.

## Figures and Tables

**Table 1:** Common solid reaction mechanisms [33,34].

No.	*g*(*α*)	*f*(*α*)	Rate-Determining Model
1	1 − (1 − *α*)^2/3^	3/2(1 − *α*)^1/3^	Chemical Reaction
2	1 − (1 − *α*)^1/4^	4(1 − *α*)^3/4^	Chemical Reaction
3	(1 − *α*)^−1/2^ − 1	2(1 − *α*)^3/2^	Chemical Reaction
4	(1 − *α*)^−1^ − 1	(1 − *α*)^2^	Chemical Reaction
5	(1 − *α*)^−2^ − 1	1/2(1 − *α*)^3^	Chemical Reaction
6	*α* ^3/2^	2/3*α*^−1/2^	Nucleation
7	−ln(1 − *α*)	1 − *α*	First Order, *n* = 1
8	[−ln(1 − *α*)]^2/3^	3/2(1 − *α*)[−ln(1 − *α*)]^1/3^	Avrami–Erofeev
9	[−ln(1 − *α*)]^1/2^	2(1 − *α*)[−ln(1 − *α*)]^1/2^	Avrami–Erofeev
10	*α*	1	Contracting Disk
11	1 − (1 − *α*)^1/2^	2(1 − *α*)^1/2^	Contracting Cylinder
12	1 − (1 − *α*)^1/3^	3(1 − *α*)^2/3^	Contracting Sphere
13	*α* ^2^	1/2*α*	1-*D* Diffusion
14	*α* + (1 − *α*)ln(1 − *α*)	[−ln(1 − *α*)]^−1^	2-*D* Diffusion
15	[1 − (1 − *α*)^1/3^]^2^	(3/2)(1 − *α*)^2/3^[1 − (1 − *α*)^−1/3^]^−1^	3-*D* Diffusion
16	1 − 2*α*/3 − (1 − *α*)^2/3^	(3/2)[(1 − *α*)^−1/3^ − 1]^−1^	3-*D* Diffusion
17	[(1 + *α*)^1/3^ − 1]^2^	(3/2)(1 + *α*)^2/3^[(1 + *α*)^1/3^ − 1]^−1^	3-*D* Diffusion
18	1 + 2*α*/3 − (1+*α*)^2/3^	(3/2)[(1 + *α*)^−1/3^ − 1]^−1^	3-*D* Diffusion
19	[(1 + *α*)^−1/3^ − 1]^2^	(3/2)(1 + *α*)^4/3^[(1 + *α*)^−1/3^ − 1]^−1^	3-*D* Diffusion

**Table 2:** The *E_a_* is calculated by four methods based upon thermogravimetric data.

*α*	*E_a_* (kJ/mol)					
	FWO	DAEM	Starink	Tang	Jiao et al. [24]	Jiang et al. [12]
0.1	180.9	179.4	176.7	178.5	147.4	368
0.2	212.1	211.7	212.0	210.7	164.6	298
0.3	211.5	211.0	211.3	208.4	165.0	277
0.4	205.4	204.4	204.7	203.8	164.1	270
0.5	201.2	199.9	200.2	199.4	163.3	270
0.6	197.3	195.6	196.0	196.0	161.8	263
0.7	195.6	193.7	194.0	194.0	161.1	256
0.8	208.1	206.7	207.0	207.0	164.1	253
Average	201.5	200.3	200.2	199.7	161.4	281.9
Value ^a^	15.5%	16.1%	17.6%	16.1%	10.9%	40.8%

^a^ The value is the deviation between the maximum and minimum *E_a_* and the average *E_a_* percentage [53,54].

**Table 3:** Calculation values of *E_a_* for the CR method.

No.	*g*(*α*)	*E_a_* (kJ/mol)					
		5 K/min	20 K/min	40 K/min	60 K/min	80 K/min	Average
1	1 − (1 − *α*)^2/3^	111.2	121.5	120.1	122.4	114.5	117.9
2	1 − (1 − *α*)^1/4^	125.3	136.9	135.4	138.2	129.5	133.1
3	(1 − *α*)^−1/2^ − 1	155.1	169.2	167.7	171.4	161.0	164.9
4	(1 − *α*)^−1^ − 1	178.0	194.1	192.5	196.9	185.2	189.3
5	(1 − *α*)^−2^ − 1	230.1	250.7	249.1	255.2	240.5	245.1
6	*α* ^3/2^	157.2	171.5	169.5	172.6	161.8	166.5
7	−ln(1 − *α*)	134.6	147.0	145.5	148.5	139.4	143.0
8	[−ln(1 − *α*)]^2/3^	86.0	94.1	93.1	95.0	88.9	91.4
9	[−ln(1 − *α*)]^1/2^	61.7	67.7	66.8	68.3	63.63	65.6
10	*α*	101.1	110.5	109.1	111.1	103.8	107.1
11	1 − (1 − *α*)^1/2^	116.6	127.4	126.0	128.5	120.8	123.9
12	1 − (1 − *α*)^1/3^	122.4	133.7	132.2	134.8	126.4	129.9
13	*α* ^2^	213.3	232.6	229.9	234.1	219.7	225.9
14	*α* + (1 − *α*)ln(1 − *α*)	232.7	253.7	251.0	255.7	240.2	246.7
15	[1 − (1 − *α*)^1/3^]^2^	255.9	278.9	276.2	281.7	264.8	271.5
16	1 − 2*α*/3 − (1 − *α*)^2/3^	240.3	262.0	259.3	264.3	248.4	254.9
17	[(1 + *α*)^1/3^ − 1]^2^	193.9	211.4	208.9	212.6	199.3	205.2
18	1 + 2*α*/3 − (1 + *α*)^2/3^	200.1	218.2	215.6	219.5	205.9	210.0
19	[(1 + *α*)^−1/3^ − 1]^2^	175.8	191.8	189.3	192.5	180.4	186.0

**Table 4:** Reaction mechanisms are determined by masterplots and the iterative procedure methods.

No.	*g*(*α*)	Masterplots					ln(*g*(*α*)) vs. ln*β*	
		5 K/min	20 K/min	40 K/min	60 K/min	80 K/min	Slope	*R* ^2^
1	1 − (1 − *α*)^2/3^	3.96	5.14	1.51	4.11	9.46	0.639	0.992
2	1 − (1 − *α*)^1/4^	1.54	2.78	1.24	2.29	4.25	0.717	0.995
3	(1 − *α*)^−1/2^ − 1	11.64	0.19	0.61	0.04	1.13	0.877	0.995
4	(1 − *α*)^−1^ − 1	102.66	4.37	0.18	2.61	17.48	0.999	0.992
5	(1 − *α*)^−2^ − 1	1335.39	87.80	0.98	61.10	271.06	1.277	0.981
6	*α* ^3/2^	11.61	2.78	1.26	2.38	4.33	0.876	0.988
7	−ln(1 − *α*)	4.18	1.47	1.05	1.24	1.70	0.767	0.996
8	[−ln(1 − *α*)]^2/3^	40.12	5.94	1.57	4.63	11.30	0.511	0.996
9	[−ln(1 − *α*)]^1/2^	69.58	8.91	1.82	6.82	18.38	0.384	0.996
10	*α*	48.30	6.93	1.69	5.47	13.64	0.584	0.988
11	1 − (1 − *α*)^1/2^	23.50	4.19	1.41	3.39	7.32	0.669	0.994
12	1 − (1 − *α*)^1/3^	15.82	3.24	1.30	2.65	5.24	0.701	0.995
13	*α* ^2^	0.63	0.299	0.83	0.40	0.15	1.167	0.988
14	*α* + (1 − *α*)ln(1 − *α*)	21.53	0.31	0.50	0.17	2.78	1.274	0.992
15	[1 − (1 − *α*)^1/3^]^2^	121.99	5.37	0.14	3.41	21.38	1.401	0.995
16	1 − 2*α*/3 − (1 − *α*)^2/3^	43.19	1.17	0.37	0.67	6.53	1.316	0.993
17	[(1+*α*)^1/3^ − 1]^2^	2.92	1.44	1.08	1.35	1.75	1.059	0.984
18	1 + 2*α*/3 − (1+*α*)^2/3^	1	1	1	1	1	1.094	0.985
19	[(1 + *α*)^−1/3^ − 1]^2^	13.17	3.00	1.30	2.58	4.84	0.959	0.979
